# T-Minus 10 Days: The Role of an Academic Medical Institution in Field Hospital Planning

**DOI:** 10.1017/S1049023X21000224

**Published:** 2021-02-18

**Authors:** Sue Anne Bell, Lesly A. Dossett, Jesus Cespero, Mayuri Guntupalli, Keith Dickey, Jonathan Eliason, Dawn Coleman

**Affiliations:** 1. School of Nursing, University of Michigan, Ann Arbor, Michigan USA; 2. Medical School, University of Michigan, Ann Arbor, Michigan USA; 3. Michigan Medicine, University of Michigan, Ann Arbor, Michigan USA

**Keywords:** alternate care site, pandemic, preparedness

## Abstract

Alternate care sites (ACS) are locations that can be converted to provide either in-patient and/or out-patient health care services when existing facilities are compromised by a hazard impact or the volume of patients exceeds available capacity and/or capabilities. In March through May of 2020, Michigan Medicine (MM), the affiliated health system of the University of Michigan, planned a 500 bed ACS at an off-site location. Termed the Michigan Medicine Field Hospital (MMFH), this ACS was intended to be a step-down care facility for low-acuity COVID-19 positive MM patients who could be transitioned from the hospital setting and safely cared for prior to discharge home, while also allowing increased bed capacity in the remaining MM hospitals for additional critical patient care. The planning was organized into six units: personnel and labor, security, clinical operations, logistics and supply, planning and training, and communications. The purpose of this report is to describe the development and planning of an ACS within the MM academic medical center (AMC) to discuss anticipated barriers to success and to suggest guidance for health systems in future planning.

## Introduction

At the onset of the COVID-19 pandemic, modeling estimates placed the number of cases as far-beyond the projected availability of patient beds in many areas across the United States. Alternate care sites (ACS) are facilities that can be converted to provide health care services when existing facilities are compromised by a hazard impact or the volume of patients exceeds available capacity and/or capabilities.^1^ By the end of April 2020, up to 28 freestanding ACS ranging from 50 beds to 3,000 beds were either under construction or accepting patients.^1^

The University of Michigan (Ann Arbor, Michigan USA), through its affiliated academic medical center (AMC) Michigan Medicine (MM), began the process to stand up an ACS within its own existing facilities to be internally planned and staffed. A licensed non-profit, MM is one of the largest medical centers in the state, with 1,043 licensed beds and reporting close to 2.5 million clinic visits in 2018.^2^ Located in the greater Detroit (Michigan USA) area, one of the initial US hotspots, MM accepted a higher number of transfers requiring critical care, while also maintaining access for other programs such as trauma and burn care. The ACS was intended for low-acuity COVID-19 patients who could be transitioned from the hospital setting, allocating bed capacity in the main hospital for more critical patient care. The purpose of this report is to describe the development and planning of an ACS within the MM system, and to suggest guidance for health systems in future planning by detailing an account of the efforts for establishing future ACS.

**Figure 1.**
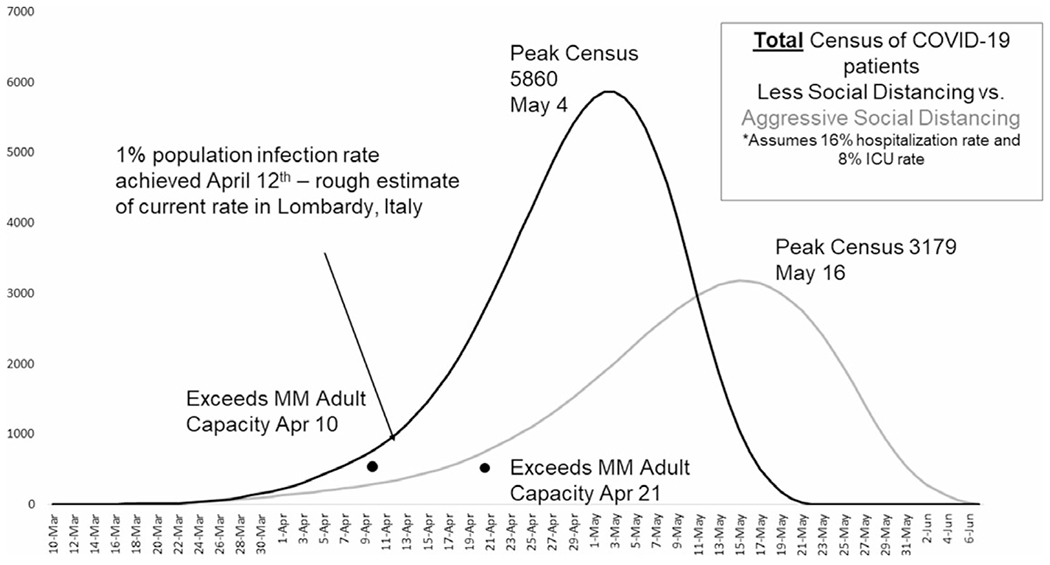
Michigan Medicine COVID-19 Surge Modeling, March 24, 2020. Abbreviations: ICU, intensive care unit; MM, Michigan Medicine.

In mid-March, forecasting models conducted at MM and updated daily (Figure 1) indicated a substantial patient surge related to COVID-19 that was expected to exceed the institution’s 1,000 bed capacity.^3^ It is important to note that these models represented early projection before refined parameters and assumptions for COVID-19 were developed. Southeast Michigan hospitals were also expected to be inundated with a surge of COVID-19 patients. Extensive preparations were undertaken at MM’s main hospital in order to convert existing space to intensive care units (ICUs) in order to accommodate the surge while also continuing to service non-COVID patients in need of critical care. Critical care capacity at MM nearly tripled during the surge from a baseline 107 adult ICU beds to 243 adult COVID-designated ICU beds and 24 non-COVID ICU care beds with the intentional transition of moderate care, Post-Acute Care Unit (PACU), and children’s hospital beds into “flex” critical care space. Despite this, modeling suggested the surge was still expected to overwhelm capacity.

Planning worked from a “T-minus” standpoint for a 500 bed ACS locally termed the “Michigan Medicine Field Hospital” (MMFH) began on March 27, 2020, with an expected date for operations to begin on April 9, 2020 (the date one day prior to MM’s bed capacity being overwhelmed based on modeling data). The goal of the ACS was to decompress the main hospital, allowing critically ill patients to remain on-site while low-acuity patients were accommodated within the ACS. Early communication indicated that regional and state resources were focused on the existing ACS already in progress elsewhere in the state.

## Report

### Organization of Planning

**Figure 2.**
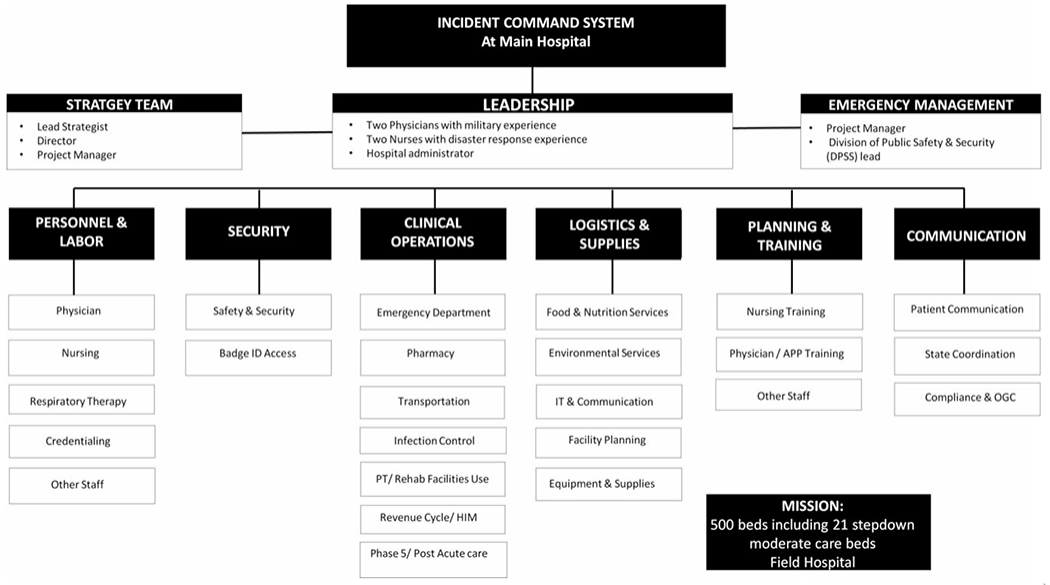
Michigan Medicine Field Hospital Organizational Structure. Abbreviations: APP, advanced practice provider; HIM, health information management; OGC, Office of Global Communications; PT, physical therapy.

A core leadership team, tasked by the MM Department of Strategy, was composed of two physicians with military experience, two nurses with disaster response experience, and one hospital administrator. Under this, organizational units were formed loosely based on the US military’s staff structure (Figure 2) of personnel and labor, security, clinical operations, logistics and supply, planning and training, and communications. This chain of command optimized communication and accountability to minimize duplication of efforts and confusion. Interdisciplinary nature was a strength of the planning process where members of the health care team, logistics, facilities management, security, emergency management, and infection control, among others, worked together in close coordination. The MMFH operated under the incident command system in place at the main hospital.

### Logistics and Supply

#### Site Selection and Planning—

**Figure 3.**
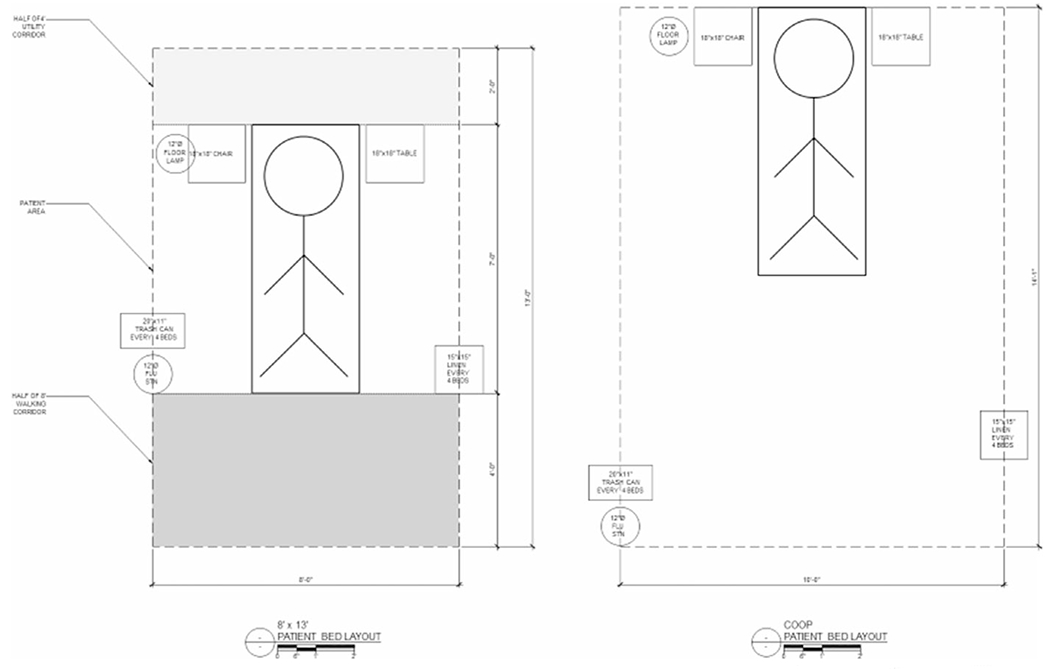
Michigan Medicine Field Hospital Draft Layout.

The first goal was to identify an off-site location, as most of MM hospital that could be converted towards patient care was already being used or planned for use. Site visits were made of indoor athletic facilities and the dormitories closest to the hospital. The University of Michigan’s newly opened 73,000 square foot indoor track and performance center facility, a twelve-minute drive from the main hospital, was ultimately selected as the location for a field hospital. This facility had a number of attributes that would facilitate effective patient care. It could provide a “clean” and a “dirty” side in order to reduce the risk of transmission and to form distinct areas of function to maintain organization. A draft layout was completed in roughly two days (Figure 3) for 519 beds, including a 20-bed higher-acuity area for decompensating patients needing transfer back to MM for care.

#### Clinical Operations—

The MMFH was designed to care for COVID-19 positive patients needing lower acuity care (defined as supportive care for patients unable to be discharged home). Any patient meeting criteria for care at MMFH care would first be seen in MM and transferred to the field hospital. Criteria were developed in collaboration with the main hospital on the types of patients to be accepted at the MMFH (Table 1). While patients were expected to be low acuity, in order to accommodate different levels of care, along with the degree of uncertainty for adjustments based on day-to-day review of projections, two units were established. The first unit was dedicated to caring for those patients that could not be discharged home but could recover with close monitoring in a field hospital setting. The second unit was for those needing “moderate” or critical care, or transfer to the main hospital. Inclusion and exclusion criteria were specified; these were defined based on staff capacity and safety reasons. The consensus was that any patient needing critical care should be at the main hospital unless all resources there were exceeded. Intake and discharge team process and flow were established. A separate code team to address immediate emergencies was also developed.

At the beginning, planning discussions centered on providing safe care in a resource-limited environment. On-site pharmacy would be in place, and was initially planned without computing support, where supplies would be in the “dirty” patient care area without a Pyxis. Processes grew more standardized as the length of time to stand up the field hospital was extended. For example, with increased time, a plan was designed to use electronic health records, instead of paper charting as initially planned, as well as capability for phlebotomy and radiology were added. However, these processes also added complexities in planning that posed potential challenges to implementation.

The goal was to provide the highest level of comprehensive care possible in a temporary setting. Aspects of comfort or well-being provided in a hospital, such as televisions and visitors, could not be accommodated due to risk of transmission. Additional challenges arose in the face of decisions on how best to allocate staffing and personal protective equipment (PPE) resources with regard to convalescence versus acute care. Because of the nature of COVID-19, it was assumed that the majority of patients would be older adults, many with co-morbidities and mobility issues. Decision-making for the MMFH revolved around how to meet the rehabilitation needs of these patients, including physical therapy (PT) and physical medicine and rehabilitation (PM&R), and the associated PPE needed for those staff.

Standardized processes were needed to mobilize the large number of staff needed to care for the expected surge of up to 500 patients. These were developed in a rapid, but team-based manner (Table 2). Physician process development occurred in close collaboration with hospitalists at the main hospital. A clinical “field manual” (ie, operational guide) was developed that specified MMFH criteria and care processes. Michigan Medicine based nurses with military or other relevant experience were recruited as nurse leads to develop the care processes. This team spent two weeks developing the processes and flow seen in Table 2.

#### Procurement—

Sourcing of supplies to set up the field hospital was a large issue. The surge of COVID-19 patients combined with the rapid scale-up of field hospitals and ACS at multiple places across the country meant essential supplies—outside of PPE—such as cots, linens, and privacy screens were difficult or impossible to source. Items were out of stock or no longer available for purchase, as huge competition existed regionally and beyond. The PPE shortages were an on-going concern, as was the case throughout the United States. In order to begin providing care, it had to be understood if there was enough PPE to safely staff the MMFH. Part of this was thinking about the amount of time staff could safely be in PPE inside the patient care area and how to minimize staff who needed to be in the “dirty” zone in order to conserve PPE.

#### Communications—

A communications lead was designated for patients and families, within the university, and at the local, state, and federal level. Discussions with the local and state emergency managers and regional health care coalitions were conducted through the communications lead and relayed to the larger team in daily meetings. At the patient and family level, the communications lead collaborated with the Office of Patient Experience (OPE) to ensure proper communication mechanisms for patients and family members as patients are transitioned to the MMFH. The OPE also worked on collecting donations such as iPads, chargers, and toiletries to distribute to patients at MMFH. All media requests and community inquiries were addressed by MM’s public relations department in keeping with the larger incident command system in place.

#### Staffing—

The MMFH was designed to be an extension of the MM hospital where resources were envisioned to come primarily from existing staff. With this model, credentialing, background checks, and familiarity with MM systems and processes would already be in place. One major advantage of an AMC-run facility is physician staffing capacity. Since academic physicians generally are not 100% clinical, MM physicians could be tapped to provide extra clinical capacity, as well as resident trainees. Individual resilience of staff to thrive in a challenging operational environment, COVID risk status, and personal issues (such as dual health care provider household or single parents) were also considered. Safe staffing ratios were determined by group consensus from the MMFH leadership team, in conjunction with the MM nursing administration. Because MM was only providing essential services at the time, staffing estimates were able to safely support the field hospital.

## Discussion

### Anticipated Barriers and Gaps to Implementation

Among the barriers were communication at the state governmental level, issues with staffing and supplies, and challenges with the shifting deadline to open the field hospital. Initially, the plan was to stand up the field hospital without significant state government involvement while still following state regulations. As the days progressed, shifting deadlines and changing modeling generated concerns about when, and later if, to stand up the field hospital. As guidance and communication from levels of government rapidly changed, the team needed increased flexibility to take these adjustments in stride and to mitigate internal issues such as planning team misalignment and duplicative efforts.

Barriers around supplies and staffing were also a concern. Projected staffing gaps and staff education needs became apparent. The main hospital needed to meet priority ancillary staff resources, which may have a limited staff of some types to be able to be redeployed to the MMFH. Further, this staff and other types of staff had limited training in using PPE or working in a COVID-19 positive environment, while also considering potential exposure; at this early stage of the pandemic, testing capacity was limited. Finally, obtaining adequate supplies was also a significant challenge, as shortages of all types were present across the country. With this knowledge, MM was respectful of the conservation of PPE as a critical resource.

### Strengths of University-Based Facility

A number of strengths were identified. The ACS being situated within an existing AMC meant that there were numerous staff with clinical expertise, and tapping into those faculty and staff with prior military or disaster experience was critically important. The use of internal resources in terms of staff, stuff, and structure meant that those involved already had some level of familiarity with each of these. Tremendous volunteerism was also observed from MM staff of all types, with numerous offers to redeploy to the MMFH or to task-shift into needed roles in order to meet staffing needs. The team-based strategy to develop processes was a strength, as was its interdisciplinary make-up. This enabled work on processes separately, then refined together. Finally, the organizational structure put in place at the beginning of the planning process allowed for clear communication and a line of responsibility.

It is worth noting the limitation that since the MMFH was developed internally, there may have been other resources that were not aware of that could have been used or that could have been pooled with other institutions.

### Impact of the Michigan Experience for Future ACS Planning

Optimizing MM’s ability to “surge in place” by preparing to stand up an ACS allowed for the ability to uphold occupational safety and standards of care.^4^

The best possible outcome was achieved, and by April 16,2020, the decision was made that the MMFH would not be operationalized in the scope of the current outbreak. Standing up the field hospital in a second wave, however, remains a possibility. Efforts are continued to understand and improve the health care delivery system in a pandemic, including effective functioning when augmented with an ACS in a surge situation (Table 3).^5^ For example, as planning was underway, the US Army Corps of Engineers conducted a site visit to assess construction of the MMFH, and discussions were underway. Future ACS at MM may include support from the Army Corps.

## Conclusion

Although this experience is specific to COVID-19, much of what has been learned is generalizable to other events requiring a field hospital. While COVID-19 continues to critically affect the United States, and with more surges expected in the coming months,^6^ careful surge capacity and capability planning around ACS is clearly a need for AMCs. As health systems around the country address the continued influx of COVID-19 patients, it is essential to understand the aspects of ACS in order to serve the projected needs of a community.

## Figures and Tables

**Table 1.** Patient Eligibility Criteria for Transfer to Michigan Medicine Field Hospital

Eligible (meets ALL criteria after 48 hours of hospitalization)	Ineligible
O2 requirement <6L/min nasal cannula	Actively managed acute complications of COVID (eg, AKI, COPD exacerbation)
Deterioration index ≤55	Actively managed acute non-COVID conditions (eg, severe hyponatremia)
Clinically stable or improving in past 24 hours	Requires acute specialty or surgical care
Medically dischargeable but with disposition issues (eg, SAR/SNF, nursing home)	Continuous cardiac monitoring (telemetry)
Patients requiring End of Life/Hospice Care (>24 hours life expectancy)	Need for intermittent hemodialysis/peritoneal dialysis
	Tracheostomy
	Requiring additional isolation (eg, C. diff)
	Weight >300lbs

Abbreviations: AKI, acute kidney injury; COPD, chronic obstructive pulmonary disease; C. diff, clostridium difficile colitis; SAR, subacute rehabilitation; SNF, skilled nursing facility.

**Table 2.** MMFH Clinical Operations Processes Developed

Clinical Operations Processes Developed
Just-in-Time Training
Staff Orientation
Donning and Doffing Procedure
Code Team
Intake and Transfer
Discharge Planning
Care Delivery
Patient and Staff Food and Nutrition
Medical Records
Nursing Documentation
Physician Documentation
Computing and IT
Infection Control and PPE
Respiratory Therapy
Patient Care Experience
Staff Flow into Clean and Dirty Areas
Pharmacy
Physical Therapy and Rehabilitation
Radiology and EKG
Laboratory and Phlebotomy
Bed Management
Morgue
Patient Orders

Note: Not an exhaustive list.

Abbreviations: EKG, electrocardiogram; MMFH, Michigan Medicine Field Hospital; PPE, personal protective equipment.

**Table 3.** When Health Care Systems Should Consider Establishing an ACS

Consider establishing an ACS if “yes” to any/all of the following questions:
• Does your jurisdiction or health care system have an anticipated or current need for additional surge capacity or capability?
• Does your jurisdiction or health care system have the ability to support additional non-traditional facilities as part of the health care system?
• Does your jurisdiction or health care system have the ability to staff an additional health care site using traditional or non-traditional medical providers?

Source: Assistant Secretary for Preparedness and Response.^1^

Abbreviation: ACS, alternate care site.

## Abbreviations:

ACS: alternate care site
AMC: academic medical center
ICU: intensive care unit
MM: Michigan Medicine
MMFH: Michigan Medicine Field Hospital
OPE: Office of Patient Experience
PPE: personal protective equipment
PT: physical therapy
